# Experience Sharing: Final Year MBBS Journey

**DOI:** 10.31729/jnma.6363

**Published:** 2021-04-30

**Authors:** Sujata Maharjan, Ashmita Pandey

**Affiliations:** 1 Kathmandu Medical College and Teaching Hospital, Sinamangal, Kathmandu, Nepal

**Keywords:** *COVID-19*, *medical student*, *stress*

## Abstract

Studying medicine is tough. The saying: It is hard to enter medical school but easier later is a myth. All the semesters and years have their trials and tribulations but the final year is known among students to be a terror. Here we share our experience of the final year hoping it could give insight to the medical students about what to expect in the ultimate year and prepare themselves mentally as well as academically beforehand.

## INTRODUCTION

Studying medicine is tough. The saying: It is hard to enter medical school but easier later is a myth. It's not only about studying, but also about meeting new people, adjusting to the new environment, and networking with people.^1^ It is about taking the responsibility for someone's life into your hands. It is considered one of the most challenging undergraduate courses.^2^ All the semesters and years have their trials and tribulations but the final year is known among students to be a terror and rightly so. On the flip side, it also gave us unforgettable memories for a lifetime. Here are some of our experiences.

### 1. Academic burden

The curriculum for the final year with four major and their allied subjects is very vast. Most of the topics had been covered in the 5^th^ and 6^th^ semester. But since we had been freshly transitioning from basic science to clinical, the first 12 months went by in a blink of an eye. Later, it became difficult to cover everything in the last few months. At the last moment, going through the deep approach was not the right option and When it comes to university final exams, the surface apathetic approach is more likely to fail. So, to pass the University exam the strategic approach was the only way to gain the best grades possible.^3^ The past questions and important topics as told by teachers and seniors became our savior. It would have been preferable if we had started using the deep approach, which focuses on comprehending concepts and connecting ideas, 3 from the beginning of our clinical life. Instead of cramming the day before the test, try to prepare smartly and on a regular basis. Make a regular routine for yourself and stick to it.

### 2. Psychological Stress

The incidence of psychological illnesses including anxiety, burnout, and depression is high among medical students.^4^ The common reasons for high stress and anxiety included the pressure of passing exams, the pressure of living up to family's expectations, fear of stepping into the real world of medicine.^5^ Furthermore, the long-term lockdown due to COVID-19 pandemic caused further worsening in the psychological and learning behaviors. We remember saying “I just want to pass, I just want this final year to get over”

### 3. Sleepless Nights

Many nights were spent awake, particularly during the exams. Medical students may not consider sleep to be a top priority due to their academic obligations, but sleep deprivation may affect their academic performance and on the other hand, maybe a cause, symptom, or comorbidity of stress or psychiatric disorders.^6^ A day between tests seemed insufficient to recover from the previous day's fatigue and prepare for the next one. There was no concentration in the studies, and no restful sleep. Medical students are well aware that mindfulness meditation and aerobic exercise may be beneficial in reducing stress,^7^ however, we couldn't afford to devote time to meditation and exercise at the last minute of the exam.^6^

### 4. Lack of enough clinical exposure

“To study the phenomena of disease without books is to sail an uncharted sea, while to study books without patients is not to go to sea at all.” Sir William Osler 8 Due to the COVID-19 pandemic and the lockdown imposed, our practical classes were limited to about a month of virtual classes and 2 months of physical classes that were more of a group discussion, case scenarios and patient exposure was inadequate. There were months of major practical postings in the fifth, sixth and eighth semester which were not fully utilized and everything was left to be learned in the final semester. Medical student lectures on a real patient in a clinical environment are needed to account for the doctor-patient relationship, empathy, compassion, and communication skills, all of which account for the humanistic principles of medicine. Hence online teaching sessions and simulated virtual patient case scenarios could be one of the ways of learning during such a crisis but could not be a replacement of actual learning with the patients.^9^

### 5. Break in the momentum due to lockdown

The eighth-semester went by fearing and figuring out the way to get through the year. End semester internal examinations were a real wake up call to start becoming serious. The ninth semester kicked off with taking every opportunity to study, discuss with friends, and practice, being attentive in classes, asking questions and preparing mentally and academically for the finals. The momentum had only just been picked, when the COVID-19 pandemic happened. Consequently, the college minimized gatherings by suspending all campus activities including suspension of classroom teaching and switching to online classes to decrease the rapid spread of the virus. Lockdown came with several challenges to all medical students such as the uncertainty in course completion, examination, the fear of exposure to COVID-19, the lack of guidance and the loss of learning opportunities. The situation remains unclear due to the COVID-19 and no one knows when the lockout will be imposed again. It can be difficult to stay motivated during a lockdown in such circumstances. It is important to stay motivated and make the most of your time. We can do this by making a schedule and sticking to it. It can also be overwhelming, so we can use workouts and yoga to help us cope. Discipline is crucial.

### 6. Virtual classes and exams

The unprecedented global disruption and curricular restructuring prompted by the pandemic led to the introduction of a novel method of delivering education to medical students. Online teaching platforms were beneficial due to their worldwide accessibility, ensuring that all medical students regardless of their current location were able to access webinars as they happen or record for later.^10^ However, there were some demerits: lagging audios, hours of waiting for teachers to join, the struggle to get early to the class before zoom maxed out and rejoin after a session is over. The transition to online medical education also saw a change in examination methods with the online pre university examination. Such an examination format raises legitimate concerns over the honesty and fairness of the process.

### 7. The Eleventh hour: Struggle to manage time

A study suggests that almost half of the undergraduate medical students fail to efficiently manage study time and spread the workload evenly throughout the academic year.^11^ When the college reopened after 6 months of lockdown, trying to catch up on uncovered topics along with revising the other, learning too many things in too little time, made the days very hectic and stressful.

### 8. Friendship

It is very difficult to get through medical school, mostly the final year alone. Social support from friends and family are shown to have an inverse relationship with the prevalence of depression and burnout in the medical student population.^12^ The tea talks, classroom dramas, group studies, and zoom sessions with a great group of friends we are surrounded with kept us sane.

### 9. That doctor feeling

Medical education is long and physically and emotionally demanding.^4^ It feels like you have been studying for so long and yet you are so far from becoming a doctor. But, it is this year that gave us the signal of being close to the destination.

### 10. Knowing our faculties better

Teachers are the backbone of an education system. They play a dynamic role in the learning of students and have a significant influence on the delivery of healthcare through the students they teach once they are in medical practice.^13^ A teacher is not only a role model who has an influence on every facet of students' growth and on developing their innate potential but is also a motivator, guide, and friend.^14^ Being blessed with valuable knowledge, the stories of success and struggles, inspirational lessons from the faculties is something to treasure for life.

### 11. Clinical postings

The best way to learn critical clinical knowledge, professionalism, and humanistic patient interactions are at the bedside.^15^ Most medical students have positive responses and learning attitudes towards different aspects of bedside teaching.^16^ This is the stepping stone before the journey of patient care and medical service begins. Although short, the time spent in the wards in the eighth semester, the joy when a patient called us doctor, the smiles in the face of the patient when we greeted and thanked them, the respect in their eyes, the grin on their face when they lied to us about not smoking or drinking alcohol, the times we made a mistake and got scolded by the doctor in the rounds or during bedside classes, the case we presented to the teacher, the times we got it right have been imprinted in our minds for a lifetime.

### 12. Bright side of Lockdown

Though academically hurt, we were presented with a moment to stop and breathe, time to reconnect with family, space to grow personally and learn new skills, an opportunity to attend many national and international seminars, workshops and conferences virtually which would not have been possible without lockdown.

### 13. Being confident and competent

The final year presented us with a feeling of competence and confidence. Although lacking in exposure and practice, the knowledge, the inspiration gained implanted in us a belief of being ready to enter into the real world of patient care.

### 14. On the last day, the whole class was together

The day the batch photo was taken, was probably the first time the whole class was together since the lockdown and the last time it will be. The environment of the class was electric, loud and still so soothing, just like a family reunion.

## WAYS FORWARD

The final year of MBBS is the most challenging and also the most crucial learning phase. Academic burden, the stress of examination, race against time could make the journey arduous. It is very easy to lose focus and feel like you are on the edge. However, there are so many good things to look forward to. Yes, you have to devote yourself fully to your studies, but if you study regularly and smartly, it should not trouble you much. Rather than cramming a day before exams, try to study daily. Plan your time wisely, make daily routines and stick to them. It can be exhausting and overwhelming at times but gradually you will acclimatize. Try to keep a balance between your studies and daily life. Take time to de-stress yourself. It is very important not to panic and to be kind to yourself. Know your limits and work hard. Never hesitate to ask for help from your friends, family, teachers and seniors. Remember to spend as much time as possible in the ward with the patients, because ultimately what matters in the exams and also as a doctor is how good you are with your patients and how well you can converse with and comfort them.
